# PCRRT Expert Committee ICONIC Position Paper on Prescribing Kidney Replacement Therapy in Critically Sick Children With Acute Liver Failure

**DOI:** 10.3389/fped.2021.833205

**Published:** 2022-02-02

**Authors:** Rupesh Raina, Sidharth K. Sethi, Guido Filler, Shina Menon, Aliza Mittal, Amrit Khooblall, Prajit Khooblall, Ronith Chakraborty, Harsha Adnani, Nina Vijayvargiya, Sharon Teo, Girish Bhatt, Lee Jin Koh, Chebl Mourani, Marcelo de Sousa Tavares, Khalid Alhasan, Michael Forbes, Maninder Dhaliwal, Veena Raghunathan, Dieter Broering, Azmeri Sultana, Giovanni Montini, Patrick Brophy, Mignon McCulloch, Timothy Bunchman, Hui Kim Yap, Rezan Topalglu, Maria Díaz-González de Ferris

**Affiliations:** ^1^Cleveland Clinic Akron General Medical Center, Akron, OH, United States; ^2^Department of Nephrology, Akron Children's Hospital, Akron, OH, United States; ^3^Kidney and Renal Transplant Institute, Medanta, The Medicity Hospital, Gurgaon, India; ^4^Division of Paediatric Nephrology, Department of Paediatrics, Western University, London, ON, Canada; ^5^Division of Pediatric Nephrology, Department of Pediatrics, Seattle Children's Hospital, University of Washington School of Medicine, Seattle, WA, United States; ^6^Department of Pediatrics, All India Institute of Medical Sciences, Jodhpur, India; ^7^Akron Nephrology Associates, Akron, OH, United States; ^8^Department of Medicine, Northeast Ohio Medical University, Rootstown, OH, United States; ^9^Anne Arundel Medical Center, Annapolis, MD, United States; ^10^Khoo Teck Puat-National University Children's Medical Institute, National University Hospital, Singapore, Singapore; ^11^Department of Pediatrics, ISN-SRC, Pediatric Nephrology, All India Institute of Medical Sciences (AIIMS), Bhopal, India; ^12^Department of Paediatric Nephrology, Starship Children's Hospital, Auckland, New Zealand; ^13^Pediatrics, Hôtel-Dieu de France Hospital (HDF), Beirut, Lebanon; ^14^Pediatric Nephrology Unit, Santa Casa de Belo Horizonte, Belo Horizonte, Brazil; ^15^Pediatric Nephrology, King Saud University College of Medicine, Riyadh, Saudi Arabia; ^16^Department of Pediatric Critical Care, Akron Children's Hospital, Akron, OH, United States; ^17^Department of Pediatric Critical Care, Institute of Liver Transplantation and Regenerative Medicine, Medanta, The Medicity, Gurgaon, India; ^18^Klinik für Allgemeine und Thoraxchirurgie, Universitätsklinikum Schleswig-Holstein, Kiel, Germany; ^19^Department of Pediatric Nephrology, Dr. M R Khan Shishu Hospital & Institute of Child Health, Dhaka, Bangladesh; ^20^Pediatric Nephrology, Dialysis and Transplant Unit, Fondazione Istituto di Ricerca e Cura a Carattere Scientifico Cà Granda, Ospedale Maggiore Policlinico, Milan, Italy; ^21^Department of Clinical Sciences and Community Health, University of Milan, Milan, Italy; ^22^Department of Pediatrics, University of Rochester School of Medicine, Rochester, NY, United States; ^23^Red Cross War Memorial Children's Hospital, University of Cape Town, Cape Town, South Africa; ^24^Pediatric Nephrology and Transplantation, Children's Hospital of Richmond, Virginia Commonwealth University (VCU), Richmond, VA, United States; ^25^Department of Pediatrics, Yong Loo Lin School of Medicine, National University of Singapore, Singapore, Singapore; ^26^Department of Pediatric Nephrology, School of Medicine, Hacettepe University, Ankara, Turkey; ^27^Department of Pediatrics, University of North Carolina School of Medicine, Chapel Hill, NC, United States

**Keywords:** pediatric, PALF, acute liver failure (ALF), acute kidney injury (AKI), ALF

## Abstract

Management of acute liver failure (ALF) and acute on chronic liver failure (ACLF) in the pediatric population can be challenging. Kidney manifestations of liver failure, such as hepatorenal syndrome (HRS) and acute kidney injury (AKI), are increasingly prevalent and may portend a poor prognosis. The overall incidence of AKI in children with ALF has not been well-established, partially due to the difficulty of precisely estimating kidney function in these patients. The true incidence of AKI in pediatric patients may still be underestimated due to decreased creatinine production in patients with advanced liver dysfunction and those with critical conditions including shock and cardiovascular compromise with poor kidney perfusion. Current treatment for kidney dysfunction secondary to liver failure include conservative management, intravenous fluids, and kidney replacement therapy (KRT). Despite the paucity of evidence-based recommendations concerning the application of KRT in children with kidney dysfunction in the setting of ALF, expert clinical opinions have been evaluated regarding the optimal modalities and timing of KRT, dialysis/replacement solutions, blood and dialysate flow rates and dialysis dose, and anticoagulation methods.

## Introduction

The management of acute kidney injury (AKI) in the setting of acute liver failure (ALF) can be challenging, especially in the pediatric population. Current guidelines in the treatment of kidney dysfunction secondary to liver failure includes conservative management, intravenous fluids, and kidney replacement therapy (KRT), based primarily on adult literature (1). There is a lack of randomized controlled trials in the pediatric subset to design a diagnostic algorithm. Together, the Pediatric Continuous Renal Replacement Therapy (PCRRT) Workgroup and the International Collaboration of Nephrologists and Intensivists for Critical Care Children (ICONIC) provide clinical practice points for children with AKI in the setting of ALF.

## Methodology

PubMed, CINAHL, EMBASE, Cochrane, Web of Science, and Google Scholar were used to search the literature based on the PICO model regarding AKI in PALF. After screening, only eight relevant studies (one prospective, one case-control, and six retrospective studies) with 196 patients aged 0–18 years were included (2–9). A quality assessment tool graded the literature. Due to the paucity of literature regarding the treatment of AKI in PALF, select manuscripts by Hanudel et al. and Spinale et al. in the treatment of neonatal hyperammonemia were also referenced. After rigorous Delphi surveys, educated consensus statements were then formulated to guide the clinician in the management of AKI in PALF (Table 1). These practice points have been developed in conjunction with the PCRRT workgroup and are designed to provide provisional, time-sensitive answers, based on the best available evidence to questions related to AKI in PALF. The comprehensive methodology can be referenced in Supplementary Materials 1–6.

**Table 1:** Summary and practice points.

	**Summary and practice points**		**Grading**
Introduction	1	ALF is defined by biochemical evidence of liver injury, absence of known pre-existing chronic liver disease, coagulopathy unable to be corrected by Vitamin K administration, and an INR >1.5 if the patient is encephalopathic, or INR >2 if not.	2C
		AKI in liver failure is indicated by an increase in SCr of ≥0.3 mg/dl (≥26.5 μmol/L) over a 48-h period or a percentage increase in SCr of ≥50% from baseline over a 7-day period.	2C
		Diagnosis of HRS is indicated by an absolute increase in SCr. ≥0.3 mg/dl within 48 h, urinary output ≤ 0.5 ml/kg, or a percent increase in SCr ≥50% using the last available value of outpatient SCr within 3 months as the baseline value.	2C
		We suggest patients be referred to critical care when (1) any grade of encephalopathy is reached, (2) oliguria, hypotension, respiratory distress, or extra-hepatic organ failure occurs, (3) INR>4.0 which requires exchange transfusion.	2C
Conservative management of AKI in ALF	5	There are limited studies on albumin, vasoconstrictors, and vaptans in the pediatric population, but literature regarding their proven efficacy in adults is applicable.	3B
		Albumin infusions at have shown to be potentially effective in volume expansion in AKI.	3B
		The panel suggests that a vasopressin infusion should be considered if noradrenaline >1 mcg/kg/min.	3C
		Large-volume paracentesis in the setting of respiratory compromise or fluid overload may be followed by an albumin infusion.	3C
		If patient is in need of ongoing resuscitation with fluids, inotropes, and/or vasopressors, the panel suggests a 1–2 mg/kg/dose of hydrocortisone administered every 6 h. It is recommended in ALF in multi-organ failure with relative adrenal insufficiency.	3C
		A urine output >1 ml/kg/h should be maintained with a mean blood pressure stabilized >50th percentile.	3B
		The panel suggests that ventilation pressures and FiO_2_ should be adjusted to stabilize PaCO_2_ levels between 4.5 and 5.2 K_pa_ and mean arterial saturations at more than 96%.	3C
		Suggested nutritional requirements: • Non-ventilated patients are suggested to have a caloric intake of 120–150% of the recommended allowance. Ventilated patients should refer to the Schofield equation with parameters outlined by age and sex. • Carbohydrate, fat, and protein intake is suggested to be based on age, ranging from 6 to 13 g/kg/day, 1–4 g/kg/day, and 1–2 g/kg/day, respectively.	3C
		The panel suggests the administration of 100 mg/kg/day of N-acetyl cysteine in all patients as a continuous infusion.	3C
KRT indications	6	Cystatin-C estimates of eGFR are the most accurate in pediatric ALF with AKI.	3C
		KRT is suggested to be initiated in children with the following conditions: • Electrolyte and Metabolic abnormalities (resistant to fluid therapy) § Metabolic acidosis § Hyponatremia (<130 meq/L), Hyperkalemia, Hypermagnesemia, Hyperphosphatemia § Uremia with bleeding § Elevated lactate level • Hepatic encephalopathy (Grade 3–4) • Ammonia >150 μmol/L and uncontrolled or >200 μmol/L • Fluid overload o Severe hemodynamic instability o Pericarditis • Increased ICP following the failure of mannitol and hypertonic saline treatment	3C
		KRT can be initiated in AKI with associated multi-organ dysfunction as well as low Pediatric Risk of Mortality (PRISM) scores	3C
		We suggest using CKRT, rather than IHD, for AKI patients with acute brain injury due increased intracranial pressure or generalized brain edema.	3C
		Patients with liver and kidney dysfunction are more susceptible to hyperammonemia which may ultimately lead to cerebral edema.	3B
		EEG may be used to assess neurological statis and subclinical seizures in PALF.	3C
Modalities of kidney replacement therapy	7	The literature on the use of CKRT's in PALF with AKI is limited, but positive.	3B
		CKRT is suggested over IHD due to the slower rate of solute removal, gradual correction of hyponatremia, hemodynamic stability, and risk in increasing intracranial pressure.	3C
Machines and circuits for KRT	S18	CKRT machines may be able to modify blood and dialysate flow rates according to the child's weight.	3C
		In neonates, the total extracorporeal blood volume may exceed 10% (unlike pediatrics) with the use of crystalloids, colloids, or packed RBCs to prime the circuit.	3C
		High flux dialyzers can be applied if administering hemofiltration and should account for the body weight and surface area of the patient.	3C
Vascular access	S18	In pediatric patients undergoing KRT, the right internal jugular vein is the ideal catheter insertion site for children <20 kg or if the catheter is <10 F.	3C
		Large diameter catheters should be utilized for safety and efficiency with a reduced risk of complications.	3C
Dialysis/replacement solution	9	Lactate free dialysate solution may be utilized in the pediatric population with ALF.	3C
		The dialysis fluid contents can be modified to benefit the patient's needs.	3C
		Increased concentration of calcium and sodium can support hemodynamic stability.	3C
Blood and dialysate flow rate and dialysis dose	9	The rate of blood flow in children ≤ 30 kg undergoing KRT should be within 3-5 ml/kg/min. For neonates, a blood flow rate of 50–80 ml/min should be utilized. For patients >30 kg, the blood flow rate is suggested to be gradually increased to 150–200 ml/min and dialysate flow rate set at 500 ml/min. The dialysis flow rate can increase to double the blood flow rate, but the dialysis flow rate may not be >300 ml/min when administering IHD.	3C
		The panel suggests using high-volume CKRT between 60 and 120 mL/kg/h, depending on ammonia clearance as well as the clinical and biochemical response.	3C
Anticoagulation	9	In children with ALF requiring KRT, the decision to use anticoagulation can be determined by an individual's risk assessment. For patients with no contraindications to RCA, its use in ALF is recommended over unfractionated heparin.	3C
		The panel suggests that a patient's platelet count should be stabilized above 50,000/ml, especially in patients on CKRT.	3C
		The panel suggests that coagulopathies should not be corrected unless the patient is actively bleeding or an invasive procedure has be conducted.	3C
		Use of heparin, prostacyclin, citrate, or no anticoagulants are all potential options dependent on drug availability, cost, and trained manpower—specifically when using regional citrate anticoagulation.	3C
		The panel suggests that a thromboelastography should be performed on patients with refractory bleed.	3C
		The panel suggests 40 mcg/kg of Recombinant Factor VII be administered if bleeding is unable to be controlled by FFP, platelets, and/or cryoprecipitate.	3C
		Anticoagulant administration requires monitoring of the activated partial thromboplastin time or Activated Clotting Time (ACT) with a target ACT of 180–220 s. The ideal APTT is 10 s over the baseline to 1.5 times the regular value.	3C
Initiation, duration, and monitoring	10	Early initiation of KRT is suggested, especially as a bridge to liver transplantation in critically ill pediatric patients with ALF.	3C
		The length and frequency of a dialysis session depends on the volumetric needs of the patient and hemodynamic stability.	3C
		Non-invasive monitoring of blood pressure and regular assessment of serum B.U.N./creatinine, intake and output, daily weight change, and extended serum electrolytes are essential in caring for pediatric patients with AKI and underlying ALF.	3C
		A combination of CKRT, MARS, and TPE may be used for treatment in AKI with ALF	3C
		A combination of CKRT, MARS, and TPE may be used for treatment in AKI with ALF o High-volume CKRT is suggested for treating ALF, maintaining fluid balance, recovering metabolic function, and removing water soluble toxins. o MARS is suggested for hepatic encephalopathy o TPE is suggested for coagulopathy	3C

## Definition

The Pediatric ALF Study Group (PALFSG) defines ALF in the pediatric population as (1) biochemical evidence of liver injury, (2) absence of known pre-existing chronic liver disease, (3) coagulopathy not corrected by vitamin K administration, and (4) an International Normalized Ratio (INR) >1.5 if the patient has encephalopathy or >2.0 if the patient does not (1, 10).

An increase in SCr ≥0.3 mg/dl (≥26.5 μmol/L) over a 48-h period or a percentage increase in SCr ≥50% from baseline over a 7-day period is indicative of AKI in ALF (11–13). AKI can be based on the pRIFLE (Pediatric Risk, Injury, Failure, Loss, End Stage Renal Disease) and KDIGO (Kidney Disease Improving Global Outcomes) criteria (Supplementary Material 7). The etiology of AKI in ALF can be classified into (A) functional (pre-renal) causes; (B) intrinsic causes including acute tubular necrosis; or (C) Hepatic Renal Syndrome (HRS) (14). Clinical indications for HRS includes an absolute increase in SCr. ≥0.3 mg/dl within 48 h, urinary output ≤ 0.5 ml/, or a percent increase in SCr ≥50% using the last available value of outpatient SCr within 3 months as the baseline value. The 2015 International Club of Ascites consensus definition of AKI in patients with cirrhosis or HRS uses the KDIGO criteria (Supplementary Material 8).

## Incidence and Mortality of AKI in ALF

In our analysis, a total of 5 studies were included that provided information on AKI epidemiology in pediatric ALF (Table 2). The pooled proportion (95% CI) of AKI incidence among ALF patients was 36.23% (18.76–55.82%) [*I*^2^: 97.14% (95.30–98.25%); *p* <0.0001; random effect model; 5 studies; *n* = 1,144] (Supplementary Materials 9, 10) (19–23). Among AKI patients with ALF, the pooled proportion (95% CI) of mortality was 28.86% (8.10–56.00%) [*I*^2^: 83.93% (51.70–94.65%); *p* = 0.0020; random effect model; 3 studies; *n* = 203] (Table 3) (19, 21, 22). Visual inspection of the funnel plot and Egger test show a symmetrical distribution indicating no evidence of publication bias (Supplementary Materials 11, 12). Factors for AKI in ALF include decreased cardiac output, loss of intravascular volume, reduced afterload, inflammation, non-selective beta-blockers, vasodilators, non-steroidal anti-inflammatory drugs, or angiotensin-converting enzyme inhibitors (16).

**Table 2:** Meta-analysis of proportion of AKI among ALF patients across different studies.

**Study**	**Event/sample size**	**Estimate (95% CI) %**	**Random weight (%)**
**Pediatric incidence of AKI in ALF**			
Bluhme et al. (15)	9/51	17.65 (8.40–30.87)	19.3
Deep et al. (16)	19/84	22.62 (14.20–33.05)	20.1
Spinale et al. (8)	29/34	85.29 (68.94–95.05)	18.5
Gonwa and Wadei (17)	175/392	44.64 (39.65–49.72)	21.0
Lahmer et al. (18)	102/583	17.50 (14.50–20.83)	21.1
Total (random effects)	334/1,144	36.23 (18.76–55.82)	100

*The analysis included AKI incidence among AKI patients with ALF across different studies. Heterogeneity across studies was quantified using the I^2^ statistic, and the I^2^ > 50 % indicated significant heterogeneity. The fixed-effect analytical model was used to pool the results of studies with acceptable or no heterogeneity, while the random-effect model for results of studies with significant heterogeneity. A total of 5 studies were included. The pooled proportion (95% CI) of AKI incidence among ALF patients was 36.23% (18.76–55.82%) [I^2^: 97.14% (95.30–98.25%); p < 0.0001; random effect model; 5 studies; number of patients = 1,144]*.

**Table 3:** Meta-analysis of proportion of mortality among AKI patients with ALF across different studies.

**Study**	**Event/sample size**	**Estimate (95% CI) %**	**Random weight (%)**
**Pediatric mortality of AKI in ALF**			
Bluhme et al. (15)	2/9	22.22 (2.81–60.01)	27.2
Deep et al. (16)	10/19	52.63 (28.86–75.55)	32.8
Gonwa and Wadei (17)	26/175	14.86 (9.94–21.01)	40.0
Total (random effects)	38/203	28.86 (8.10–56.00)	100

*The analysis included AKI mortality among AKI patients with ALF across different studies. Heterogeneity across studies was quantified using the I^2^ statistic, and the I^2^ > 50 % indicated significant heterogeneity. The fixed-effect analytical model was used to pool the results of studies with acceptable or no heterogeneity, while the random-effect model for results of studies with significant heterogeneity. Among AKI patients with ALF, the pooled proportion (95% CI) of mortality was 28.86% (8.10–56.00%) [I^2^: 83.93% (51.70–94.65%); p = 0.0020; random effect model; 3 studies; number of patients = 203]*.

## Conservative Management of AKI in ALF

Conservative interventions include use of albumin, vasoconstrictors, and vaptans (24).

Albumin levels indicate the staging of fibrosis and prognosis in chronic liver disease in children *via* the ischemia-modified albumin (IMAR) (25). Evidence suggests that albumin levels should be monitored in pediatric patients since it's effective in reducing post-paracentesis, circulatory dysfunction, and mortality from AKI in adults (16, 26–28). However, there is lack of data on the efficacy of albumin in specifically pediatric AKI and this topic should be further studied in the pediatric population to recognize the utility of albumin infusions in children.

The synthetic vasopressin analog terlipressin has shown potential as an ideal V1-receptor mediated vasoconstrictor capable of decreasing portal blood flow and therefore, increasing blood flow to the kidney. Its use in pediatrics was evaluated (*n* = 16) and an improvement in SCr at 24 h (*p* = 0.386) with an increase in urine output in the HRS-AKI subgroup was observed (29). Unfortunately, terlipressin is currently not available in the United States or Canada and data on the use of other vasopressors such as octreotide are not available.

Vaptans such as Tolvaptan have an agonist effect on V1 receptors, causing an increase in plasma vasopressin levels which can lead to vasoconstriction. The efficacy and safety of Tolvaptan in refractory ascites and LVP in adults is applicable to pediatrics. Practice points regarding conservative management for pediatrics are provided in Table 1, section Conservative management of AKI in ALF.

## Kidney Replacement Therapy Indications

The indications for the use of KRT in PALF (*n* = 45) have been reported as oligo-anuria (31%), hyperammonemia (29%), hepatic encephalopathy (27%), high lactate (22%), fluid overload (13%), resistant metabolic acidosis (7%), resistant hyperkalemia (2%), and hyponatremia (2%) (2). The baseline characteristics of PALF with AKI (*n* = 19) compared to just PALF (*n* = 65) shows a correlation with: (1) higher baseline bilirubin (mean difference (MD) AKI vs. no AKI: 8.5 mg/dl, 95% CI 3.3–13.8, *p* = 0.002), (2) higher INR (MD AKI vs. no AKI: 0.98, 95% CI: 0.1–1.8, *p* = 0.029), (3) higher Model for (Pediatric) End-Stage Liver Disease (M(P)ELD) (MD AKI vs. no AKI: 5.9, 95% CI: 1.5–10.3, *p* = 0.009), (4) higher incidence of systemic inflammatory response (OR 10.4, 95% CI: 2.7–39.6, *p* <0.0005) and (5) higher incidence of spontaneous bacterial peritonitis (OR 8.4, 95%CI: 1.4–50.2, *p* = 0.022) (19). The risk factors for AKI in pediatrics is a baseline bilirubin >17.7 mg/dL (adjusted OR: 1.07; 95% CI: 1.008–1.135, *p* = 0.025) in combination with systemic inflammatory response syndrome (adjusted OR: 8.659; 95% CI: 2.18–34.37, *p* = 0.002) (19). These findings are supported with a case-control study on KRT in pediatric liver transplant patients (*n* = 32) (3). At listing, KRT recipients (*n* = 8) were found to have greater M(P)LED scores (26 vs. 16, *p* = 0.02), increased bilirubin (31.8 vs. 9.4, *p* = 0.006), increased creatinine (2.55 vs. 0.36, *p* = 0.01), decreased glomerular filtration rate (GFR: 21 vs. 102, *p* < 0.001), and decreased platelets (53 vs. 128, *p* = 0.001) (3). 100% of the patients (*p* = 0.03) had ascites, spontaneous bacterial peritonitis in 50% (*p* = 0.02), gastrointestinal bleeding in 100% (*p* = 0.01), infections in 88% (*p* = 0.01), and toxic levels of vancomycin in 38% (*p* = 0.01) (3). Although the KRT group had lower liver and kidney function at baseline, the long-term kidney function was comparable between the patients with HRS receiving KRT and the control group without HRS. It was concluded that pediatric patients with HRS, including infants, benefited from KRT (*p* <0.05) (3).

Hyperammonemia is the strongest indicator for the use of KRT in PALF. Pediatric patients with ALF and/or AKI are prone to hyperammonemia due to their impaired metabolism and/or excretive processes. The primary distinction of hyperammonemia in infants and children involves urea cycle disorders (UCDs) and organic acidemias, also referred to as inborn errors of metabolism. Hyperammonemia is defined as >100 μmol/l (170 μg/dl) in neonates or ≥50 μmol/l (85 μg/dl) in term infants, and children (7). Ammonia levels >150 μmol/L should be promptly evaluated as an increased risk of morbidity and mortality is present, especially if elevated levels of ammonia (>800 μmol/L) persist for >24 h. Hanudel et al. and Spinale et al. each assessed two cases of pediatric hyperammonemia and found such elevated levels (~800 μmol/L) required CKRT (8, 9). Patients with ALF have also reported cases of cerebral edema where hyperammonemia is thought to be the primary pathogenic driver (4). Increased intracranial pressure in ALF with early neurocritical care has been observed to drastically reduce mortality (5). Cerebral edema in ALF can also worsen during seizures where cerebral oxygen requirements exacerbate its condition. Continuous forms of veno-venous hemofiltration and/or dialysis, such as CKRT, can cause a more gradual change in plasma osmolarity while maintaining cardiovascular stability (6).

The accurate monitoring of GFR, especially in PALF, is important in detecting a decline in renal function and promptly initiating treatment. GFR can be measured by s-creatinine and/or p-cystatin C or by inulin and or/iohexol clearances (15). A comparison of all variations of GFR measurements in 91 children found that p-cystatin C-based formulas as well as s-creatinine-based formulas were the most accurate at 84–87.5%, least bias at 0.19–4.0 ml/min/1.73 m^2^, and least misclassified at 24.7–25% (15). Cystatin-C based formulas were even more accurate and less biased than creatinine-based formulas in patients with renal function <75 ml/min/1.73 m^2^ (15). Practice points can be found in Table 1, section Kidney Replacement Therapy Indications.

## Modalities of Kidney Replacement Therapy

Initiation of KRT is needed when conservative management fails to achieve the desired outcomes in AKI patients. The use of CKRT or intermittent hemodialysis (IHD) is considered based on the patient's hemodynamic condition. Due to constant fluid shifts with ALF, CKRT is superior due to slower solute removal, better hemodynamic stability, a more gradual correction of hyponatremia, and a lower likelihood in worsening intracranial pressure (17). CKRT is also effective in maintaining fluid balance, recovering metabolic function, and removing water soluble toxins associated with the development of AKI, HRS, and an exacerbated of hepatic injury (18). CKRT is also able to remove inflammatory cytokines, which have been associated with the development of AKI, HRS, and ALF (18). Alternatively, successful usage of sustained low efficiency dialysis (SLED) in place of CKRT has been reported in adults (30). In cases where CKRT is not available or where vascular access is not possible, PD may be used as a last resort. When considering the modality of KRT, hemodynamic stability, severity of illness, and patient preferences should be considered.

In a study by Deep et al., PALF patients with AKI (*n* = 45) who were treated with CKRT observed a 58% (*n* = 26) survival rate, 42% (*n* = 19) successfully bridged to liver transplantation, and 17% (*n* = 7) spontaneously recovered (2). Even among PALF patients who did not receive a liver transplant, CKRT significantly improved survival (HR: 4; 95% CI: 1.5–11.6; *p* = 0.006) (2). Overall, the use of CKRT in reducing hyperammonemia by 48 h after initiation drastically increased survival (HR, 1.04; 95% CI, 1.013–1.073; *p* = 0.004) (2). A similar cohort of PALF with AKI (*n* = 344) also found that an overall survival rate of 58% for those on CKRT (31). Significant improvements in hemodynamic stability and neurological status in children with ALF on high-volume CKRT was also reported (32). Practice points are provided in Table 1, section Modalities of Kidney Replacement Therapy. Additional background information regarding the machines and circuits for KRT and optimal vascular access can be found in Supplementary Material 13.

## Extracorporeal Liver Support Devices (Eclads)

Monitoring patients with ECLADs, such as the Molecular Adsorbents Recirculation System (MARS) and Single-Pass Albumin Dialysis (SPAD), are necessary to optimize a patient's condition before transplantation. Both techniques mimic excretory, synthetic, and metabolic functions of the liver, allowing for removal of water insoluble/protein-bound substances *via* high-flux membranes using an albumin rich dialysate (17, 18).

MARS technology combines CKRT with the removal of large protein-bound particles *via* albumin dialysis. Used in tandem, MARS is able to remove albumin-bound substances which accumulate during liver failure *via* recirculated album-enriched dialysate combined with a charcoal filter and an ion exchange resin. MARS is used as bridging procedure to transplantation if the patient's INR >3 and for one of the following conditions: (1) hepatic encephalopathy ≥ grade II, (2) creatinine values >3.5 mg/dl and oliguria <0.5 ml/kg body weight/h or (3) Hepatorenal syndrome (HRS) (33). Nadlin et al. observed a pediatric cohort (*n* = 5) who were initiated on MARS as a bridging procedure to liver transplantation (33). All 5 patients had poor prognostic factors, 4 had hepatic encephalopathy ≥3 and needed ventilation support, 3 were on vasopressive agents, and 2 had cerebral edema (33). Patient survival and graft survival were 100 and 80%, respectively, without sequelae (33).

Prometheus is a form of albumin dialysis and combines fractionated plasma separation, adsorption, and hemodialysis (34). It differs from MARS as it enables the direct contact of the patient's albumin with the adsorbing materials through filtration of the albumin fraction and sent through a secondary circuit containing two adsorber columns. Its primary advantage is that it relies on endogenous over exogenous albumin.

SPAD is a form of hemodiafiltration with non-recirculated albumin dialysis, which uses a filter identical to MARS to eliminate albumin-bound toxins. However, MARS is safer due to the removal of the stabilizers and higher clearances for water-soluble substances such as ammonia, cytokines, creatinine, and urea because of the high dialysate flow rates (35). It should be noted that MARS is also preferred due to financial constraints as the albumin in SPAD may not always be economically accessible; SPAD requires large amounts of albumin which is often wasted whereas MARS recirculates and recycles albumin. The key differences are highlighted in Supplementary Material 14 with the corresponding circuits in Supplementary Materials 15–18. Despite the limited data, MARS shows promise in pediatric ALF for the removal of bilirubin and bile acids which CKRT fails to remove.

Therapeutic plasma exchange (TPE) in ALF patients removes protein bound toxins and corrects coagulopathies (36). It can work better than blood products, such as fresh frozen plasma (FFP), which carry a risk of volume overload, worsening hepatic encephalopathy, and hypocalcemia. TPE is fluid neutral and avoids an exogenous protein load (36). An improved clinical response has been anecdotal at best, but no significant survival rates have been reported.

## Prescription of Kidney Replacement Therapy in Liver Failure

### Dialysis/Replacement Solution

Dialysate or replacement solution that are conventionally used typically contains sodium, potassium, calcium, chloride, magnesium, bicarbonate, and glucose (37). Lactate containing solutions are not recommended in patients with liver dysfunction because the liver is not able to metabolize it resulting in hyperlactemia and high anion gap metabolic acidosis (38).

In a study by Deep et al., a lactate-free electrolyte predilution replacement, Accusol 35, was used in all filtration episodes (2). The solution components had a pH of 7.0–7.5 and contained glucose (5 mmol/L), sodium (140 mmol/L), calcium (1.75 mmol/L), magnesium (0.5 mmol/L), and bicarbonate (35 mmol/L). The osmolality of the solution was 300 mmol/L (2). Chevret et al. used the commercially available bicarbonate-buffered replacement fluid with a similar composition: sodium (140 mmol/L), chloride (109.5 mmol/L), bicarbonate (32 mmol/L), and calcium (1.75 mmol/L) (32). Additional potassium (3.5 mmol/L) was administered in replacement fluid to prevent hypokalemia (32). We have provided practice points in Table 1, section Prescription of Kidney Replacement Therapy in Liver Failure regarding replacement solutions.

### Blood and Dialysate Flow Rate and Dialysis Dose

A marginally increased survival rate in PALF patients was observed where a high dose of CKRT was initiated (HR, 0.96, 95%CI, 0.916–1.007; *p* = 0.095) (2). Children receiving high flow hemofiltration with blood flow rates of 100–300 mL/min (dependent on the dialysis catheter size) succeeded in achieving a filtration fraction of 20–25% without premature clotting of the dialysis circuit (32). A target blood flow rate of 3–5 ml/min/kg body weight in children with AKI and end-stage liver disease was also documented (39). It should be noted that for neonates, a blood flow rate of 50–80 ml/min should be utilized (40). The blood flow rates applied by Deep et al. ranged from 50 to 250 mL/min according to weight (Supplementary Material 19) (41). Similarly, in IHD for <6 h, the blood flow rate is suggested to begin at 3 ml/kg/min and progress to 5 ml/kg/min in the following sessions. Modifications may be necessary in order to prevent a sudden decrease in plasma urea, avoiding large disturbances in osmolality, and preventing cerebral edema (42).

Spinale et al. and Hanudel et al. each examined the use of high-dose CKRT to treat neonates with hyperammonemia (8, 9). Spinale et al. utilized a dialysis flow rate of 8,000 ml/h/1.73 m^2^ and observed a decrease in ammonia levels to <400 μmol/L within 3 h then to <100 μmol/L within 10 h (8). Hanudel et al. utilized a preliminary dialysate flow rate of 40,000 ml/hr/1.73 m^2^ to rapidly decrease ammonia levels before shifting to 4,000 ml/h/1.73 m^2^ to prevent a rebound in ammonia levels. Both reports found that rapid ammonia reduction, without any rebounds, was accomplished in a single run of CKRT.

In a study by Kreuzer et al., it was found that a dialysis post-filter infusion rate of 1,00 ml/1.73 m^2^/h during CVVH was associated with enhanced efficacy and decreased mortality in children undergoing orthotopic liver transplant (39). PALF was suggested to be treated with high volume CKRT at 60–120 mL/kg/h, depending on the ammonia clearance and clinical status (43). A linear correlation in ammonia clearance was observed when comparing the CVVH hemodiafiltration (CVVHDF) doses of 35 and 90 ml/kg/h. The limited data currently shows that the higher the dose of CKRT, the better the ammonia clearance in patients with liver failure (44).

High-volume hemofiltration (HVHF) in children is defined as ultrafiltrate flow >80 mL/kg/h (32). HVHF was initiated in patients with grade III hepatic encephalopathy with a median flow of 119 mL/kg/h (range, 80–384). After 24 h of HVHF treatment, an increase in mean arterial pressure (*p* = 0.0002), decrease in serum creatinine (*p* = 0.0002), and a decline in the grade of hepatic encephalopathy was observed (32). Additionally, HVHF promoted clearance of all inotropic and vasopressor medications, highlighting the need for special attention toward these patients. Total mortality was 45.4% (*n* = 22) with 8 pediatric patients requiring an emergency liver transplant and 5 spontaneously recovering their liver function. Overall, neurologic function and patient hemodynamics were improved in PALF prior to liver transplant (32). Various practice points have been provided in Table 1, section Prescription of Kidney Replacement Therapy in Liver Failure.

### Anticoagulation

#### Heparin

Heparin is the most commonly used anticoagulant in ALF patients receiving KRT. Kreuzer et al. documented the use of 5–25 IU/kg/h unfractionated heparin for anticoagulation without an initial heparin bolus in children undergoing dialysis prior to liver transplantation (39). Abstaining from the administration from an initial heparin bolus allowed for tight control on the activated coagulation time (ACT) for the patients on CVVH which led to no severe complications being caused by heparin (39).

#### Prostacyclin

Prostacyclin is a platelet inhibitor often used as an anticoagulant in CRRT. Goonasekera et al. found an extended duration of mean circuit life of 53 h with no complications in 31 PALF cases who received prostacyclin as an anticoagulant in 62 of 98 filtration episodes (45). The remaining 36 of 98 filtration episodes were either with heparin or with no anticoagulant. Therefore, prostacyclin may be used for bleeding or where heparin is contraindicated; it can be used alone or in combination with heparin (43, 45).

#### Citrate

Citrate is another commonly used anticoagulant as the absence of systemic anticoagulation, prolonged filter life, and a significant reduction in the risk of bleeding makes it an advantageous anticoagulant (46). Since ALF is associated with impaired citrate metabolism, it is pertinent that the rate of the calcium infusion matches the citrate dosing to prevent toxicity. Chadha et al. applied citrate in 5 pediatric patients undergoing CKRT and observed no bleeding incidents with all patients recovering kidney function (20). They concluded that citrate in children is a feasible, effective, and safe form of anticoagulation and corrective clearance (CVVH) alone is sufficient to provide citrate clearance and prevent its toxic accumulation (20). Similarly, Rodriguez et al. evaluated CKRT in 51 pediatric liver failure patients and found that regional citrate anticoagulation was effective through its long filter lives and low incidence of clotting. However, adverse events and toxicity should be carefully monitored, particularly in liver failure patients (47). Practice points are provided in Table 1, section Prescription of Kidney Replacement Therapy in Liver Failure.

## Initiation, Duration, and Monitoring

Due to high-risk complications of ALF and the excellent tolerability of the procedures, early initiation is recommended, particularly in patients exhibiting rapid disease progression. Deep et al., analyzed the use of CKRT in 45 critically ill children with ALF and found that the time to initiate CKRT from the PICU admission was lower in survivors compared to non-survivors (HR: 0.96; 95% CI: 0.916–1.007; *p* = 0.095) (2). When coupled with high-dose CKRT, an increased rate of survival was seen within 14 days (HR: 3; 95% CI: 1.0–10.3; *p* = 0.063). Deep et al. found that the median time to initiate CKRT in pediatric ALF survivors was lower in comparison to non-survivors (15.8 ± 3.0 vs. 32.4 ± 6.9 h; *p* = 0.023), which favored early initiation of KRT (2).

The duration of therapy can be guided by ammonia clearance, improvement of hepatic encephalopathy, or raised intracranial pressure. The goal to reduce ammonia within 48 h should be set. For cases involving a bridge to transplantation therapy, the duration may be guided by allograft availability. However, children may transition to IHD if the hemodynamic status permits.

Non-invasive intravascular monitoring has been shown to be effective in reducing dialysis associated morbidity in comparison to a control population (48). Electrolyte abnormalities may also occur due to the continuous mechanism of CKRT. Notably, decreased potassium, phosphate, and magnesium levels can lead to alterations in neuromuscular physiology and subsequent difficulty in weaning patients off the ventilator (48).

A combination of techniques may be used to target the water soluble and protein bound toxins of AKI in PALF, A pediatric study by Arikan et al. achieved an overall survival/discharge rate from the hospital in 73% of patients (*n* = 15) utilizing a combination of high-flux CKRT for hyperammonemia, MARS for hepatic encephalopathy, and TPE for coagulopathy (Supplementary Material 20) (49). Practice points are provided in Table 1, section Initiation, Duration, and monitoring.

## Drug Dosing Adjustments

Adjustment in drug dosages based on individual needs is a crucial aspect of providing efficient KRT. However, since data on drug doses adjusted to the modality in use (HD or CKRT) are lacking, dosage should be decided in accordance with GFR. The dose in HD is usually adjusted to a GFR <10 ml/min/1.73 m^2^ and is administered after dialysis, while those undergoing CKRT are adjusted to a GFR of 10–50 ml/min/1.73 m^2^. Extracorporeal clearance should be considered regarding the type of membrane, protein binding, and residual kidney function when calculating dosage. The fractional extracorporeal clearance should also account for hepatic, metabolic, and residual kidney clearance (50). Reviewing current medications and/or therapeutic drug monitoring to determine individualized treatment goals is essential (7). Current methods heavily rely on drug monitoring and close observation on serum concentrations, response to therapy, and toxicity.

## Conclusion

The application of CKRT in the pediatric population suffering from ALF is growing and may be valuable in patients with AKI and underlying liver failure. The available evidence forms a significant limitation for the provision of evidence-based guidelines; however, current expert recommendations can provide clinicians with valuable information on approaching pediatric patients with AKI and underlying ALF. Table 1 provides a summary of recommendations, which can be beneficial to future researchers of CKRT in pediatric patients with ALF.

## Author Contributions

All authors listed have made a substantial, direct, and intellectual contribution to the work and approved it for publication.

## Conflict of Interest

The authors declare that the research was conducted in the absence of any commercial or financial relationships that could be construed as a potential conflict of interest.

## Publisher's Note

All claims expressed in this article are solely those of the authors and do not necessarily represent those of their affiliated organizations, or those of the publisher, the editors and the reviewers. Any product that may be evaluated in this article, or claim that may be made by its manufacturer, is not guaranteed or endorsed by the publisher.

## Abbreviations

ACLF: acute on chronic liver failure
AKI: Acute kidney injury
ALF: Acute liver failure
CKRT: Continuous kidney replacement therapy
HRS: Hepato-renal syndrome
KRT: Kidney replacement therapy
SPAD: Single pass albumin dialysis
TPE: Therapeutic plasma exchange.
