# Corrigendum: PCRRT Expert Committee ICONIC position paper on prescribing kidney replacement therapy in critically sick children with acute liver failure

**DOI:** 10.3389/fped.2022.1002287

**Published:** 2022-08-12

**Authors:** Rupesh Raina, Sidharth K. Sethi, Guido Filler, Shina Menon, Aliza Mittal, Amrit Khooblall, Prajit Khooblall, Ronith Chakraborty, Harsha Adnani, Nina Vijayvargiya, Sharon Teo, Girish Bhatt, Lee Jin Koh, Chebl Mourani, Marcelo de Sousa Tavares, Khalid Alhasan, Michael Forbes, Maninder Dhaliwal, Veena Raghunathan, Dieter Broering, Azmeri Sultana, Giovanni Montini, Patrick Brophy, Mignon McCulloch, Timothy Bunchman, Hui Kim Yap, Rezan Topalglu, Maria Díaz-González de Ferris

**Affiliations:** ^1^Cleveland Clinic Akron General Medical Center, Akron, OH, United States; ^2^Department of Nephrology, Akron Children's Hospital, Akron, OH, United States; ^3^Kidney and Renal Transplant Institute, Medanta, The Medicity Hospital, Gurgaon, India; ^4^Division of Paediatric Nephrology, Department of Paediatrics, Western University, London, ON, Canada; ^5^Division of Pediatric Nephrology, Department of Pediatrics, Seattle Children's Hospital, University of Washington School of Medicine, Seattle, WA, United States; ^6^Department of Pediatrics, All India Institute of Medical Sciences, Jodhpur, India; ^7^Akron Nephrology Associates, Akron, OH, United States; ^8^Department of Medicine, Northeast Ohio Medical University, Rootstown, OH, United States; ^9^Anne Arundel Medical Center, Annapolis, MD, United States; ^10^Khoo Teck Puat-National University Children's Medical Institute, National University Hospital, Singapore, Singapore; ^11^Department of Pediatrics, ISN-SRC, Pediatric Nephrology, All India Institute of Medical Sciences (AIIMS), Bhopal, India; ^12^Department of Paediatric Nephrology, Starship Children's Hospital, Auckland, New Zealand; ^13^Pediatrics, Hôtel-Dieu de France Hospital (HDF), Beirut, Lebanon; ^14^Pediatric Nephrology Unit, Santa Casa de Belo Horizonte, Belo Horizonte, Brazil; ^15^Pediatric Nephrology, King Saud University College of Medicine, Riyadh, Saudi Arabia; ^16^Department of Pediatric Critical Care, Akron Children's Hospital, Akron, OH, United States; ^17^Department of Pediatric Critical Care, Institute of Liver Transplantation and Regenerative Medicine, Medanta, The Medicity, Gurgaon, India; ^18^Klinik für Allgemeine und Thoraxchirurgie, Universitätsklinikum Schleswig-Holstein, Kiel, Germany; ^19^Department of Pediatric Nephrology, Dr. M R Khan Shishu Hospital & Institute of Child Health, Dhaka, Bangladesh; ^20^Pediatric Nephrology, Dialysis and Transplant Unit, Fondazione Istituto di Ricerca e Cura a Carattere Scientifico Cà Granda, Ospedale Maggiore Policlinico, Milan, Italy; ^21^Department of Clinical Sciences and Community Health, University of Milan, Milan, Italy; ^22^Department of Pediatrics, University of Rochester School of Medicine, Rochester, NY, United States; ^23^Red Cross War Memorial Children's Hospital, University of Cape Town, Cape Town, South Africa; ^24^Pediatric Nephrology and Transplantation, Children's Hospital of Richmond, Virginia Commonwealth University (VCU), Richmond, VA, United States; ^25^Department of Pediatrics, Yong Loo Lin School of Medicine, National University of Singapore, Singapore, Singapore; ^26^Department of Pediatric Nephrology, School of Medicine, Hacettepe University, Ankara, Turkey; ^27^Department of Pediatrics, University of North Carolina School of Medicine, Chapel Hill, NC, United States

**Keywords:** pediatric, PALF, acute liver failure (ALF), acute kidney injury (AKI), ALF

In the published article, there was a sentence missing in Table 1, the original and corrected sentences are provided as below.

It was changed from “If patient is in need of ongoing resuscitation with fluids, inotropes, and/or vasopressors, the panel suggests a 1–2 mg/kg/dose of hydrocortisone administered six times per hour.” to “If patient is in need of ongoing resuscitation with fluids, inotropes, and/or vasopressors, the panel suggests a 1–2 mg/kg/dose of hydrocortisone administered every 6 h. It is recommended in ALF in multi-organ failure with relative adrenal insufficiency.”

The authors apologize for this error and state that this does not change the scientific conclusions of the article in any way. The original article has been updated.

## Publisher's note

All claims expressed in this article are solely those of the authors and do not necessarily represent those of their affiliated organizations, or those of the publisher, the editors and the reviewers. Any product that may be evaluated in this article, or claim that may be made by its manufacturer, is not guaranteed or endorsed by the publisher.

