# Supplementary material for: PCRRT Expert Committee ICONIC Position Paper on Prescribing Kidney Replacement Therapy in Critically Sick Children With Acute Liver Failure
Source: Front Pediatr. 2022 Feb 2;9:833205. doi: 10.3389/fped.2021.833205 (PMC8849201; doi:10.3389/fped.2021.833205)
Supplement: Supplementary file 1 [file Data_Sheet_1.zip › Supplement 1.docx]

**Supplement 1:** Methodology

Methods

*Search strategy*

The following library institutions helped support the literature review process: All India Institute of Medical Sciences, Jodhpur, India; Cleveland Clinic Akron General Library, and the Cleveland Clinic Florida Loop Alumni Library, Cleveland, Ohio, USA. Potential studies were identified in the PubMed/Medline, Embase, and Google Scholar databases, using a combination of keywords: “acute liver failure”, “acute on chronic liver failure”, “liver dialysis with peritoneal dialysis”, “pediatrics”, “hemodialysis, and sustained low-efficiency dialysis”, “continuous kidney replacement therapy” and “prolonged intermittent renal replacement therapy”. The full search strategy is illustrated in Figure 1.

*Eligible criteria*

The patient/problem/population; intervention; comparison/control/comparator; outcomes (PICO) model was applied to determine eligible studies via inclusion and exclusion criteria (**Supplement 2**). There were no filters applied to the search, and there were no language restrictions.

*Selection Criteria*

All data regarding patient characteristics, duration of liver failure, type of liver failure, modality/indications for KKRT and outcomes were extracted for review. Studies were included if they analyzed the outcomes of applying KKRT prior to liver transplant in patients with acute or chronic liver failure. Studies were only included if they analyzed either mortality, survival, bridge to transplantation, and/or AKI presence. Study designs included in our analysis were case-control, retrospective and prospective studies. Studies were excluded if they involved patients receiving any form of liver dialysis (**Supplement 3**). We also excluded systematic reviews, abstracts, and meta-analyses.

*Data Extraction*

Two reviewers searched the selected articles to assess abstract and full-text articles. A third independent investigator was involved in settling any disagreements in data extraction. Only relevant articles matching inclusion and exclusion criteria were considered for review and data extraction.

*Quality assessment of Studies*

We used the gold standard quality assessment tool for Observational Cohort and Cross-Sectional Studies from National Heart Lung and Brain Institute (NHLBI) (<https://www.nhlbi.nih.gov/health-topics/study-quality-assessment-tools>) to evaluate all our included studies. For each of the 14 questions in the assessment tool, the reviewer could respond as “yes”, “no”, and “not applicable/not reported/ cannot determine”; the questions were graded based on the response for each question. Studies with scores between 12-14 were considered good quality, 9-11 as fair quality, and < 9 as poor quality (**Supplement 4)**.

*Recommendation and Practice Points*

We have reviewed the literature, developed an expert panel called the Pediatric Continuous Renal Replacement Therapy (PCKRT) Workgroup through the AKI & CRRT 2018 - 23rd International Conference on Advances in Critical Care Nephrology. See **Supplement 20** for the names of the groups. The PCKRT workgroup provides the best practice advice and practice points based on clinical, scientific, and methodological expertise. The PCKRT workgroup ensures that practice points meet the methodological trustworthiness by accounting for the standards set forth by the Guidelines International Network and the National Academy of Medicine and voluntarily completes and posts standard reporting forms with each practice point in the Guidelines International Network library on the American College of Physician’s website. The practice points are not clinical guidelines, but provide provisional, time-sensitive answers, based on the available evidence to questions related to AKI in PALF. **Table 1** summarizes these practice points.
