# Supplementary material for: PCRRT Expert Committee ICONIC Position Paper on Prescribing Kidney Replacement Therapy in Critically Sick Children With Acute Liver Failure
Source: Front Pediatr. 2022 Feb 2;9:833205. doi: 10.3389/fped.2021.833205 (PMC8849201; doi:10.3389/fped.2021.833205)
Supplement: Supplementary file 1 [file Data_Sheet_1.zip › Supplement 11.docx]

**Supplement 11:** Forest plot of the meta-analysis of proportion of mortality among AKI patients with ALF across different studies. The lower diamond in the graph represents the pooled estimate

**
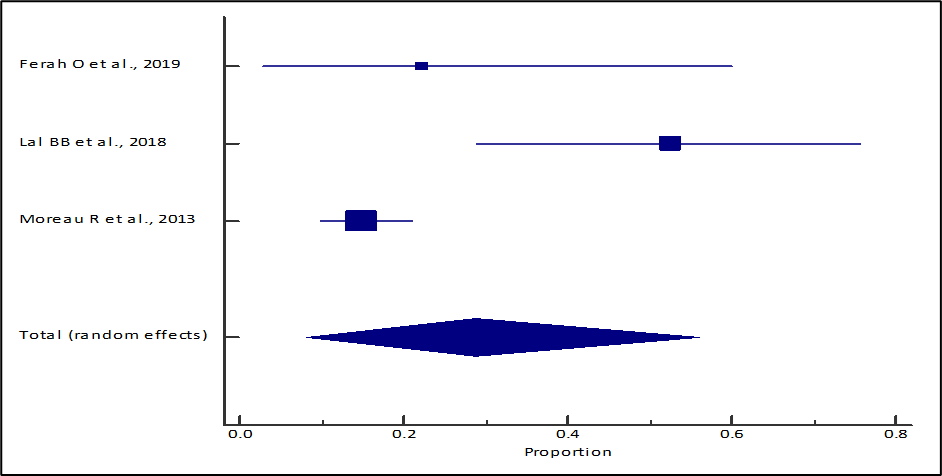
**

Supplement 11: Forest plot was used to visualize the outcomes in each study and the combined estimated outcomes with a 95% CI.
