# Supplementary material for: PCRRT Expert Committee ICONIC Position Paper on Prescribing Kidney Replacement Therapy in Critically Sick Children With Acute Liver Failure
Source: Front Pediatr. 2022 Feb 2;9:833205. doi: 10.3389/fped.2021.833205 (PMC8849201; doi:10.3389/fped.2021.833205)
Supplement: Supplementary file 1 [file Data_Sheet_1.zip › Supplement 12.docx]

**Supplement 12:** Funnel plot for showing publication bias on the mortality among AKI patients with ALF across different studies.

**
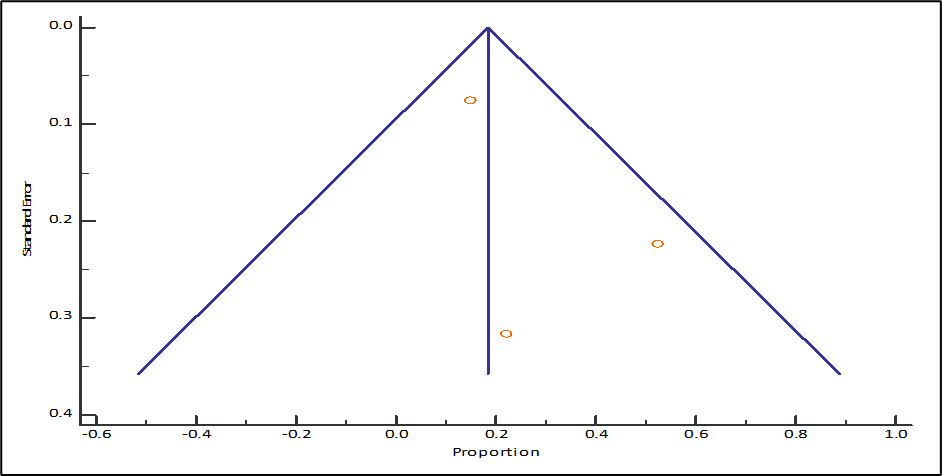
**

Supplement 12: Publication bias was assessed with a funnel plot and Egger’s test. Egger’s linear regression test was used to evaluate asymmetry. A p value <0.05 was set as the level of significance. Visual inspection of the funnel plot and Egger test (p = 0.0.4421) showed a symmetrical distribution indicating no evidence of publication bias.
