# Supplementary material for: PCRRT Expert Committee ICONIC Position Paper on Prescribing Kidney Replacement Therapy in Critically Sick Children With Acute Liver Failure
Source: Front Pediatr. 2022 Feb 2;9:833205. doi: 10.3389/fped.2021.833205 (PMC8849201; doi:10.3389/fped.2021.833205)
Supplement: Supplementary file 1 [file Data_Sheet_1.zip › Supplement 13.docx]

**Supplement 13:**

**Machines and Circuits for Kidney Replacement Therapy**

Critically, ill, unstable, and small children often require a low range of blood flow over dialysate flow, which only a small number of HD machines can provide.^57^ The appropriate machines are often classified into single-pass (Fresenius 2008, Fresenius 4008, and Fresenius 5008 series) or batch (Genius Machine from Gambro, Althin from Tina, and Diamax) dialysate.^57-60^ Alternatively, Chevret *et al.* documented the use of high-volume hemofiltration (HVHF) through a multi-filtrate CKRT.^36^

Parsons *et al.* reported the application of KRT with the Fresenius 2008K machine.^20^ In the KRT mode, polysulfone and cellulose triacetate dialyzers with citrate anticoagulation were used. While in the IHD mode, heparin anticoagulation was applied for children with HRS who underwent liver transplantation.^20^ The use of “Aquarius” (Nikkiso Europe GmbH, Hannover, Germany) has also been reported for delivering CKRT to PALF.^19^ Goonasekara *et al.* applied a similar approach for CKRT by utilizing the same technology (“Aquarius” from Edwards Life Sciences, Irvine, CA, USA) in their study which evaluated factors affecting circuit life in a similar patient population.^47^ Implementation of the Prismaflex machine with the HF20 set with positive clinical results to treat an 11-year-old with multiorgan failure (including hepatic failure), bypassing the need for the planned liver-kidney transplantation.^61^

In neonates, the total extracorporeal blood volume may exceed 10% (unlike pediatrics) with the use of crystalloids, colloids, or packed RBCs to prime the circuit.^62^ Furthermore, Deep *et al.* and Goonasekara *et al.* outlined the use of body weight as a criterion for dialyzer size. HF03 was applied to those weighing under 10 kg, HF07 was applied for patients weighing 10-50 kg, and HF1200 applied to children weighing over 50kg.^19,47^ Practice points are provided in **Table 1, S19.**

**Prescription of Kidney Replacement Therapy in Liver Failure**

A cuffed or uncuffed catheter with the size adjusted according to the child’s weight should be applied for optimal vascular access.^60^ The femoral access point is the most common site of catheter placement at 69%; however, this point is prone to catheter kinking.^63^ The right internal jugular vein is preferred an insertion site for children weighing < 20 kg (or if the catheter is <10F) due to dialysis quality, enhanced circuit survival, and its use in small caliber catheters.^42^ Recommendations on catheter sizes for pediatric patients undergoing KRT can be found in **Supplement 19.**

A cuffed or uncuffed catheter with the size adjusted according to the child’s weight should be applied for optimal vascular access.^59^ The femoral access point is the most common site of catheter placement at 69%; however, this point is prone to catheter kinking.^63^ The right internal jugular vein is preferred an insertion site for children weighing < 20 kg (or if the catheter is <10F) due to dialysis quality, enhanced circuit survival, and its use in small caliber catheters.^42^ Recommendations on catheter sizes for pediatric patients undergoing KRT can be found in **Supplement 19.**

When evaluating complications in catheter placements for dialysis in PALF, the circuit was changed in 13.3% of cases due to blockage of vascular access with a smaller access catheter contributing to a shorter circuit life.^47^ This suggests a survival advantage in using large bore catheters.^71^ Practice points can be found in **Table 1, Section S19.**
