# Supplementary material for: PCRRT Expert Committee ICONIC Position Paper on Prescribing Kidney Replacement Therapy in Critically Sick Children With Acute Liver Failure
Source: Front Pediatr. 2022 Feb 2;9:833205. doi: 10.3389/fped.2021.833205 (PMC8849201; doi:10.3389/fped.2021.833205)
Supplement: Supplementary file 1 [file Data_Sheet_1.zip › Supplement 14.docx]

**Supplement 14:** Extracorporeal Liver Support Devices (ECLADS)

*Supplement 14: SPAD: Single Pass Albumin Dialysis. MARS: Molecular adsorbent recirculating system. CVVHDF: Continuous veno-venous hemodialysis filtration. CVVHD: Continuous veno-venous hemofiltration. CWHDF: Continuous veno-venous hemodialysis filtration. CWHD: Continuous veno-venous hemodialysis.*
