# Supplementary material for: PCRRT Expert Committee ICONIC Position Paper on Prescribing Kidney Replacement Therapy in Critically Sick Children With Acute Liver Failure
Source: Front Pediatr. 2022 Feb 2;9:833205. doi: 10.3389/fped.2021.833205 (PMC8849201; doi:10.3389/fped.2021.833205)
Supplement: Supplementary file 1 [file Data_Sheet_1.zip › Supplement 15.docx]

**Supplement 15:** SPAD circuit

*Supplement 15: Single-pass albumin hemodiafiltration circuit. The albumin solution (4-5%) is passed through a high-flux filter allowing for the ultrafiltration to be replaced by substitution fluid and net ultrafiltration in volume overloaded patients*
