# Supplementary material for: PCRRT Expert Committee ICONIC Position Paper on Prescribing Kidney Replacement Therapy in Critically Sick Children With Acute Liver Failure
Source: Front Pediatr. 2022 Feb 2;9:833205. doi: 10.3389/fped.2021.833205 (PMC8849201; doi:10.3389/fped.2021.833205)
Supplement: Supplementary file 1 [file Data_Sheet_1.zip › Supplement 16.docx]

**Supplement 16**: MARS circuit

*Supplement 16: Molecular adsorbent recirculating system is a combination of a blood circuit with a polysulfone filter (A,B). This dialyses against a primary circuit of 20% albumin (C), and a conventional hemodialysis circuit to remove water soluble toxins (D). As water soluble compounds are removed from the dialysate, albumin-bound substances adhere to the charcoal filter (E) and anion exchanger (F).*
