# Supplementary material for: PCRRT Expert Committee ICONIC Position Paper on Prescribing Kidney Replacement Therapy in Critically Sick Children With Acute Liver Failure
Source: Front Pediatr. 2022 Feb 2;9:833205. doi: 10.3389/fped.2021.833205 (PMC8849201; doi:10.3389/fped.2021.833205)
Supplement: Supplementary file 1 [file Data_Sheet_1.zip › Supplement 17.docx]

**Supplement 17**: Prometheus Circuit

Supplement 17: Prometheus extracorporeal circuit. Albuflow is the albumin filter. HF: High flux. P1:First Prometheus adsorber. P2: Second Prometheus adsorber. Circles 1-3 are pressure gauges.
