# Supplementary material for: PCRRT Expert Committee ICONIC Position Paper on Prescribing Kidney Replacement Therapy in Critically Sick Children With Acute Liver Failure
Source: Front Pediatr. 2022 Feb 2;9:833205. doi: 10.3389/fped.2021.833205 (PMC8849201; doi:10.3389/fped.2021.833205)
Supplement: Supplementary file 1 [file Data_Sheet_1.zip › Supplement 18.docx]

**Supplement 18**: Therapeutic Plasma Exchange Circuit

Supplement 18: Plasma pheresis and hemodialysis can be performed together using a plasma separator and a conventional high-flux filter configured in series. Pressure gauges are placed as circles 1-4. Variations may occur depending on the systems being combined
