# Supplementary material for: PCRRT Expert Committee ICONIC Position Paper on Prescribing Kidney Replacement Therapy in Critically Sick Children With Acute Liver Failure
Source: Front Pediatr. 2022 Feb 2;9:833205. doi: 10.3389/fped.2021.833205 (PMC8849201; doi:10.3389/fped.2021.833205)
Supplement: Supplementary file 1 [file Data_Sheet_1.zip › Supplement 19.docx]

**Supplement 19:** Catheter Sizing and Blood Flow Rates for CKRT^40,41^

| **Weight  (kg)** | **Size  (French)** | **Blood Flow Rate (mls/min)** |
| --- | --- | --- |
| <5 | 6.5 | 50-80 |
| 5-15 | 8-10 | 100 |
| 15-30 | 11.5 | 150 |
| >30 | 11.5-13.5 | 200 |

*Supplement 19: Target blood flow rates based on weight and catheter size for continuous kidney replacement treatment (CKRT).*
