# Supplementary material for: PCRRT Expert Committee ICONIC Position Paper on Prescribing Kidney Replacement Therapy in Critically Sick Children With Acute Liver Failure
Source: Front Pediatr. 2022 Feb 2;9:833205. doi: 10.3389/fped.2021.833205 (PMC8849201; doi:10.3389/fped.2021.833205)
Supplement: Supplementary file 1 [file Data_Sheet_1.zip › Supplement 2.docx]

**Supplement 2:** Summary of Literature Search Process

Total articles identified =

59

Articles considered for abstract screening = 39

Excluded based on title screening = 5

Articles considered for full text review = 32

Excluded based on abstract screening = 7

Articles considered for data extraction and analysis = 8

Excluded based on full text review = 24

Wrong study population= 6

Wrong publication type = 10

Wrong study outcome = 8

Articles after duplicated removed = 44

15 duplicates were removed

Identification

Screening

Eligibility

Included
