# Supplementary material for: PCRRT Expert Committee ICONIC Position Paper on Prescribing Kidney Replacement Therapy in Critically Sick Children With Acute Liver Failure
Source: Front Pediatr. 2022 Feb 2;9:833205. doi: 10.3389/fped.2021.833205 (PMC8849201; doi:10.3389/fped.2021.833205)
Supplement: Supplementary file 1 [file Data_Sheet_1.zip › Supplement 20.docx]

**Supplement 20**: Hybrid ECLAD Protocol used at Texas Children Hospital for PALF^87^

| **Hybrid ECLAD Protocol Used at  Texas Children Hospital for PALF** | | | |
| --- | --- | --- | --- |
|  | **CKRT** | **MARS** | **TPE** |
| **Indications** | Oliguric AKI Fluid Overload Hyperammonemia | HE ≥ Grade 3  HE Grade 2 and Multiple Organ Failure | Clinical life-threatening bleeding  Medical refractory coagulopathy |
| **Modality** | CVVHDF | MARS in series with  CKRT in CVVHDF | Centrifugal TPE in tandem with CKRT |
| **Dose** | ≥3,000 mL/1.73 m²/hr | | 1.3-1.5 times plasma volume exchange  volume with FFP replacement |
| **Duration** | Continuous until recovered,  or transition to IHD | 8 continuous hrs/day  until recovery | In series with CKRT circuit as needed |

*Supplement 20: ECLAD: Extracorporeal liver-assist devices. PALF: Pediatric acute liver Failure. AKI: Acute Kidney Injury. HE: Hepatic Encephalopathy. CVVHDF: continuous veno-venous hemodiafiltration. MARS:* *Molecular Adsorbent Recirculating System. CKRT: Continuous kidney replacement therapy. TPE: Therapeutic plasma exchange. FFP: Fresh frozen plasma. IHD: Intermittent hemodialysis.*
