# Supplementary material for: PCRRT Expert Committee ICONIC Position Paper on Prescribing Kidney Replacement Therapy in Critically Sick Children With Acute Liver Failure
Source: Front Pediatr. 2022 Feb 2;9:833205. doi: 10.3389/fped.2021.833205 (PMC8849201; doi:10.3389/fped.2021.833205)
Supplement: Supplementary file 1 [file Data_Sheet_1.zip › Supplement 21.docx]

**Supplement 21:** Represented professional societies in consensus conference

| Acute Dialysis Quality Initiative <http://www.adqi.net>  American Nephrology Nurses’ Association [http://anna.inurse.com](http://anna.inurse.com/)  American Society of Nephrology [http://www.asn-online.org](http://www.asn-online.org/)  American Society of Pediatric Nephrology [http://www.aspneph.com](http://www.aspneph.com/)  Continuous Renal Replacement Therapies <http://www.crrtonline.com>  Hemolytic Uremic Syndrome (HUS) [http://www.hus-online.at](http://www.hus-online.at/index_en.html)  International Pediatric Nephrology Association [http://www.ipna-online.org](http://www.ipna-online.org/)  Indian Society of Pediatric Nephrology  www.ispnonline.org  PedsCCM: Pediatric Critical Care Nursing http://pedsccm.wustl.edu/NURSING/APN_info.html |
| --- |
