# Supplementary material for: PCRRT Expert Committee ICONIC Position Paper on Prescribing Kidney Replacement Therapy in Critically Sick Children With Acute Liver Failure
Source: Front Pediatr. 2022 Feb 2;9:833205. doi: 10.3389/fped.2021.833205 (PMC8849201; doi:10.3389/fped.2021.833205)
Supplement: Supplementary file 1 [file Data_Sheet_1.zip › Supplement 3.docx]

**Supplement 3:** Study eligibility criterion based on PRISMA guidelines

| **Criteria** | **Inclusion Criteria** | **Exclusion Criteria** |
| --- | --- | --- |
| Population | Patients with acute or chronic liver failure who received Renal Replacement therapy of any form | Studies involving patients receiving MARS, SPAD or other forms of Liver Dialysis |
| Outcomes analyzed | Mortality/ Survival  Bridge to Transplantation  AKI |  |
| Study Type | Case control studies  Prospective/Retrospective studies | Systemic Reviews  Abstracts  Meta-Analyses |

*Supplement 3: PRISMA: Preferred Reporting Items for Systematic Reviews and Meta-Analyses. MARS: Molecular Adsorbent Recirculating System. SPAD: single pass albumin dialysis. AKI: Acute kidney injury..*
