# Supplementary material for: PCRRT Expert Committee ICONIC Position Paper on Prescribing Kidney Replacement Therapy in Critically Sick Children With Acute Liver Failure
Source: Front Pediatr. 2022 Feb 2;9:833205. doi: 10.3389/fped.2021.833205 (PMC8849201; doi:10.3389/fped.2021.833205)
Supplement: Supplementary file 1 [file Data_Sheet_1.zip › Supplement 4.docx]

**Supplement 4:** Pediatrics studies that examined KRT in Liver failure

| **Study** | **N** | **Protocol** | **Dose of KRT** | **Therapy Administered** | **Outcomes** | **Complications/ Other variables** |
| --- | --- | --- | --- | --- | --- | --- |
| Deep *et al.*^19^ | 45 | Retrospective cohort- children (1-18 y) who received CKRT prior to recover or liver transplant in liver failure | Sequentially increased to 100 ml/kg/hr | CKRT- using continuous veno-venous hemofiltration  Mean Duration 54 hours | 19 Successfully bridged to transplant and 7 recovered.  Time to initiate KRT in survivors was lower than in non-survivors | Inability to reduce ammonia within 48 hrs. confers poor prognosis |
| Parsons E *et al.*^20^ | 8 | Case control study (cases n=8, Control n=24) Included 4 infants | NA | All cases started on CKRT and later transitioned to IHD | GFR at 5-year survival – 5 surviving HRS has mean GFR of 97 ml/min/1.73m^2^ compared to controls of 114 ml/min/1.73m^2^  Overall survival was lower for cases (63%) compared to control  Among infants ¼ died post-transplant, and 1 required antihypertensive at long term follow up. |  |
| Elis D *et al.*^54^ | 13 | Retrospective study | NA | Dialysis therapy in 13 of the 27 patients with renal failure (10 hemodialysis and 3 peritoneal dialysis) | Survival and recovery compared among those who received dialysis compared to those who did not-  Survival in the remaining 19 patients undergoing OLT was significantly lower compared to 114 patients with OLT and no renal failure (53% vs 81%, p less than 0.025).  the survival rate was similar whether dialysis was used or not (5/10 vs 5/9) even though the mean GFR was significantly lower in dialyzed patients (p less than 0.05).  dialysis appeared to be beneficial for the subgroup of 12 patients with HRS, 4 of whom had complete recovery of renal function after successful OLT | frequently complicated by severe gastrointestinal hemorrhage and hypotension, and directly contributed to the death of two patients prior to OLT |
| Chevret *et al.*^36^ | 22 | Retrospective observational study | Defined as UF Flow >80 ml/kg/hr,  Median dose administered was 119 ml/kg/hr | CKRT (High volume hemofiltration) | Variables (Clinical/biological) before, 24 hr and at 48 hr of High-volume hemofiltration  24 hrs: increase in mean arterial pressure (p = 0.0002) and a decrease in serum creatinine (p = 0.0002)  48 hrs: mean arterial pressure (p = 0.0005), grade of hepatic encephalopathy (p = 0.04), and serum creatinine (p = 0.0002) improved. Overall mortality was 45.4% (n =10). Emergency liver transplantation was performed in eight children. Five patients spontaneously recovered liver function. |  |
| Kreuzer *et al.*^39^ | 26/367 children listed for OLT who received Dialysis | Retrospective analysis | NA | 21 children received CVVH and 9 received Peritoneal Dailysis | Overall mortality: 77% (100% in PD group compared to 67%) in CVVH group  Urea Reduction Rate in first 24 hrs: 12.5% in PD group compared to 23% in CVVH group |  |
| Matsubara *et al.*^55^ | 16 patients( 3yr-70 yr) | Prospective Study | Filtration rate set at 500-600 ml/hr | CVVH | Change in concentration of middle molecules using High performance Liquid Chromatography: Notable removal of substances noted  8/16 had amelioration of consciousness and were weaned from hemofiltration |  |
| Rodriguez *et al.*^8^ | 51 patients | Retrospective study | minimum dose of 2000 ml/1.73m2/hour | CVVHDF | 21/51 patients received OLT; of these 10 patients were bridged to OLT on CKRT. However, 11 patients who received OLT were started on CKRT post-OLT due to primary graft nonfunction or dysfunction or hepatic artery thrombosis. 9/10 patients who were bridged to OLT on CKRT survived compared to 6/11 in the latter group. |  |
| Arikan *et al.*^56^ | 15 patients | Retrospective observational study | ≥3000 ml/1.73m2/hr | CKRT | All the patients received CKRT with hemodiafiltration for ALF or Acute on Chronic liver failure. Nine were successfully transplanted, four died prior to transplantation and two recovered spontaneously. Overall survival and hospital discharge was 73% |  |

**CKRT,** Continuous renal replacement therapy**; KRT,** Renal replacement therapy**; IHD,** Intermittent hemodialysis; **GFR,** Glomerular filtration rate**; HRS,** hepatorenal syndrome**; OLT,** orthotopic liver transplant**; UF,** ultrafiltration**; CVVH,** continuous veno-venous hemodialysis**; CVVHDF,** continuous veno-venous hemodiafiltration**; ALF,** acute liver failure
