# Supplementary material for: PCRRT Expert Committee ICONIC Position Paper on Prescribing Kidney Replacement Therapy in Critically Sick Children With Acute Liver Failure
Source: Front Pediatr. 2022 Feb 2;9:833205. doi: 10.3389/fped.2021.833205 (PMC8849201; doi:10.3389/fped.2021.833205)
Supplement: Supplementary file 1 [file Data_Sheet_1.zip › Supplement 5.docx]

**Supplement 5:** Quality assessment for all the included studies

| **Studies** | Research Question | Study population clearly specified and defined? | The participation rate of eligible persons at least 50%? | Groups recruited from the same population and uniform eligibility criteria | Sample size justification | Exposure assessed prior to outcome measurement | Sufficient timeframe to see an effect | Different levels of exposure of interest | Exposure measures and assessment | Repeated exposure assessment | Outcome measures | Blinding of outcome assessors | Follow-up rate | Statistical analysis | Overall Outcome (Good, Fair, Poor) |
| --- | --- | --- | --- | --- | --- | --- | --- | --- | --- | --- | --- | --- | --- | --- | --- |
| Deep *et al.*^19^ | Yes | Yes | Yes | Yes | Yes | Yes | Yes | Yes | Yes | Yes | Yes | N/A | Yes | Yes | 13 |
| Parsons E *et al.*^20^ | Yes | Yes | No | Yes | Yes | Yes | Yes | Yes | No | N/A | Yes | N/A | Yes | Yes | 10 |
| Elis D *et al.^54^* | Yes | Yes | Yes | Yes | Yes | Yes | Yes | Yes | No | Yes | Yes | N/A | Yes | Yes | 12 |
| Chevret *et al.^36^* | Yes | Yes | No | Yes | Yes | Yes | Yes | Yes | Yes | N/A | Yes | N/A | Yes | Yes | 11 |
| Kreuzer *et al.^39^* | Yes | Yes | No | Yes | Yes | Yes | Yes | Yes | No | No | Yes | N/A | Yes | Yes | 10 |
| Matsubara *et al.^55^* | Yes | Yes | Yes | Yes | Yes | Yes | Yes | Yes | Yes | Yes | Yes | N/A | Yes | Yes | 13 |
| Rodriguez *et al.^8^* | Yes | Yes | Yes | Yes | Yes | Yes | Yes | Yes | Yes | Yes | Yes | N/A | Yes | Yes | 13 |
| Arikan *et al.^56^* | Yes | Yes | No | Yes | Yes | Yes | Yes | Yes | Yes | Yes | Yes | N/A | Yes | Yes | 12 |

*Supplement 5: The gold standard quality assessment tool for Observational Cohort and Cross-Sectional Studies from National Heart Lung and Brain Institute (NHLBI) (*[*https://www.nhlbi.nih.gov/health-topics/study-quality-assessment-tools*](https://www.nhlbi.nih.gov/health-topics/study-quality-assessment-tools)*) to evaluate all our included studies. For each of the 14 questions in the assessment tool, the reviewer could respond as “yes”, “no”, and “not applicable/not reported/ cannot determine”; the questions were graded based on the response for each question. Studies with scores between 12-14 were considered good quality, 9-11 as fair quality, and < 9 as poor quality*
