# Supplementary material for: PCRRT Expert Committee ICONIC Position Paper on Prescribing Kidney Replacement Therapy in Critically Sick Children With Acute Liver Failure
Source: Front Pediatr. 2022 Feb 2;9:833205. doi: 10.3389/fped.2021.833205 (PMC8849201; doi:10.3389/fped.2021.833205)
Supplement: Supplementary file 1 [file Data_Sheet_1.zip › Supplement 6.docx]

**Supplement 6:** Delphi Method

**Delphi Method**

*Supplement 6: Delphi method used to arrive at the consensus opinion on our practice points.*

Multiple rounds

Practice points were finalized with the scoring sent to the panel.

A panel of nephrologists were requested to participate.

Thirty-three practice points were formulated and sent to experts.

Comments were incorporated and new practice points were added to the list and sent to the panel again.

Researchers collated scores and identified the median and IQR to identify the top practice points.

Experts added their comments on existing recommendations and proposed new ones.

They scored the practice points on a scale of 1-5; where 1 = absolutely disagree; 2 = disagree; 3 = agree; 4 = more than agree; 5 = absolutely agree.
