# Supplementary material for: PCRRT Expert Committee ICONIC Position Paper on Prescribing Kidney Replacement Therapy in Critically Sick Children With Acute Liver Failure
Source: Front Pediatr. 2022 Feb 2;9:833205. doi: 10.3389/fped.2021.833205 (PMC8849201; doi:10.3389/fped.2021.833205)
Supplement: Supplementary file 1 [file Data_Sheet_1.zip › Supplement 7.docx]

**Supplement 7:** Revised definitions for the diagnosis and staging of AKI in liver failure ^6^

| **Change of Serum Creatinine for defining AKI** | **Timeline** | |
| --- | --- | --- |
| Increase in SCr of ≥ 0.3 mg/dl (≥ 26.5 µmol/L) | 48 hours | |
| A percentage increase in SCr of ≥ 50% from baseline | 7 days | |
| Staging of AKI Stage 1 | increase in SCr of ≥ 0.3 mg/dl (26.5 μmol/L) from baseline | SCr of ≥ 1.5- to 2-fold from baseline |
| Stage 2 |  | Increase in SCr of > 2- to 3-fold from baseline |
| Stage 3 | SCr of ≥ 4.0 mg/dl(353.6 μmol/L) an acute increase of ≥ 0.3 mg/dl (26.5 μmol/L) or  initiation of renal replacement therapy | Increase of SCr of > 3-fold from baseline |
| Partial Response- | Regression of AKI Stage | a reduction of SCr to ≥  0.3 mg/dl (26.5 μmol/L) above the baseline value |
| No response | No regression of AKI |  |
| Full Response |  | Return of SCr to a value within 0.3 mg/dl (26.5 μmol/L) of  the baseline value |

*Supplement 7* ***AKI****, Acute kidney injury;* ***SCr****, serum creatinine;* ***ICA****, International Club of Ascites. Baseline SCr: a value of SCr obtained in the previous 3 months, when available, can be used as baseline SCr. In patients with more than one value within the previous 3 months; the value closest to the admission time to the hospital should be used. In patients without a previous SCr value, the SCr value on admission should be used as baseline.*
