# Supplementary material for: PCRRT Expert Committee ICONIC Position Paper on Prescribing Kidney Replacement Therapy in Critically Sick Children With Acute Liver Failure
Source: Front Pediatr. 2022 Feb 2;9:833205. doi: 10.3389/fped.2021.833205 (PMC8849201; doi:10.3389/fped.2021.833205)
Supplement: Supplementary file 1 [file Data_Sheet_1.zip › Supplement 8.docx]

**Supplement 8:** Definition of Hepatorenal Syndrome ^5^

| **Old Classification** | **New Classification** |  | |
| --- | --- | --- | --- |
| HRS- 1 | HRS-AKI | a) Absolute increase in SCr. ≥0.3 mg/dl within 48 h and/or | |
|  |  | b) Urinary output ≤ 0.5 ml/kg B.W. ≥6 h* | |
|  |  | c) Percent increase in SCr ≥50% using the last available value of outpatient SCr within 3 months as the baseline value | |
| HRS- 2 | HRS-NAKI | HRS AKD | a) e GFR<60 ml/min/1.73m^2^ for <3 months in the absence of other structural causes |
|  |  |  | b) persistent increase in SCr <50% using the last available value of outpatient SCr within 3 months as the baseline value |
|  |  | HRS-CKD | a) eGFR <60 ml/min/1.73 m^2^ for >3 months in the absence of other structural causes |

*Supplement 8:* ***HRS-1,*** *Hepatorenal Syndrome-1;* ***HRS-2,*** *Hepatorenal Syndrome-2;* ***HRS-AKI,*** *Hepatorenal Syndrome- Acute kidney Injury;* ***HRS-NAKI,*** *Hepatorenal Syndrome- Non-Acute kidney Injury;* ***HRS-AKD,*** *Hepatorenal Syndrome- Acute kidney Disease;* ***HRS-CKD,*** *Hepatorenal Syndrome- chronic kidney disease;* ***SCr,*** *Serum creatinine****; B.W.,*** *Body Weight;* ***eGFR,*** *estimated Glomerular Filtration Rate.*
